# What are the potential advantages and disadvantages of merging health insurance funds? A qualitative policy analysis from Iran

**DOI:** 10.1186/s12889-020-09417-7

**Published:** 2020-08-31

**Authors:** Mohammad Bazyar, Vahid Yazdi-Feyzabadi, Nouroddin Rahimi, Arash Rashidian

**Affiliations:** 1 Health Policy, Department of Health Promotion, Faculty of Health, Ilam University of Medical Sciences, Ilam, Iran; 2 Health Policy, Health Services Management Research Center, Institute for Futures Studies in Health, Kerman University of Medical Sciences, Haftbagh Highway, Kerman, Iran; 3 Iran Health Insurance Organization in Ilam, Ilam, Iran; 4 Information, Evidence, and Research Department, World Health Organization, Regional Office for the Eastern Mediterranean, Cairo, Egypt

**Keywords:** Health insurance, Merger, Policy analysis, Advantage, Disadvantage, Fragmentation

## Abstract

**Background:**

In countries with health insurance systems, the number and size of insurance funds along with the amount of risk distribution among them are a major concern. One possible solution to overcome problems resulting from fragmentation is to combine risk pools to create a single pool. This study aimed to investigate the potential advantages and disadvantages of merging health insurance funds in Iran.

**Methods:**

In this qualitative study, a purposeful sampling with maximum variation was used to obtain representativeness and rich data. To this end, sixty-seven face-to-face interviews were conducted. Moreover, a documentary review was used as a supplementary source of data collection. Content analysis using the ‘framework method’ was used to analyze the data. Four trustworthiness criteria, including credibility, transferability, dependability, and confirmability, were used to assure the quality of results.

**Results:**

The potential consequences were grouped into seven categories, including stewardship, financing, population, benefit package, structure, operational procedures, and interaction with providers. According to the interviewees, controlling total health care expenditures; improving strategic purchasing; removing duplication in population coverage; centralizing the profile of providers in a single database; controlling the volume of provided health care services; making hospitals interact with single insurance with a single set of instructions for contracting, claiming review, and reimbursement; and reducing administrative costs were among the main benefits of merging health insurance funds. The interviewees enumerated the following drawbacks as well: the social security organization’s unwillingness to collect insurance premiums from private workers actively as before; increased dissatisfaction among population groups enjoying a generous basic benefits package; risk of financial fraud and corruption due to gathering all premiums in a single bank; and risk of putting more financial pressure on providers in case of delay in reimbursement with a single-payer system.

**Conclusion:**

Merging health insurance schemes in Iran is influenced by a wide range of potential merits and drawbacks. Thus, to facilitate the process and lessen opponents’ objection, policy makers should act as brokers by taking into account contextual factors and adopting tailored policies to respectively maximize and minimize the potential benefits and drawbacks of consolidation in Iran.

## Background

In countries in which health insurance is the primary source of health financing, the number and size of insurance funds along with the amount of risk distribution among them are a major concern [1–3]. Health financing experts should bear in mind the degree of fragmentation in health financing as it may lead to inequity in access to health care services for different population groups.

Fragmentation should not be considered as a problem per se. However, differences among health insurance schemes in the following aspects should be taken into account by health policy makers to examine whether the situation is satisfactory. These criteria include: 1) the percent of the whole population under the coverage of each health insurance scheme; 2) the extent of differences in contribution rates, basic benefits packages, the quality of health care received by members of different risk pools; and, more importantly, 3) variations in user charges and the amount of out-of-pocket payments paid by different beneficiaries belonging to different insurance schemes for the same health services used by them [4]. The larger the gaps are, the more health financing experts should be worried about the fragmentation’s health equity impacts. One possible solution to overcome problems resulting from fragmentation is to combine risk pools to create fewer and larger ones, ideally a single pool [5, 6]. Reducing fragmentation provides more financial protection from a given level of prepaid funds, which is the critical objective of universal coverage [7].

### Health insurance system in Iran and the challenge of fragmentation

Health insurance organizations in Iran are divided into three groups according to their functions:

1. **Basic health insurance organizations:** Among such organizations are the Iran Health Insurance Organization (IHIO) [previously called the Medical Services Insurance Organization (MSIO)] with several separate sub-funds for government employees, rural residents, the self-employed and their dependents, the poor, and other groups (e.g., college students, seminary students, clergymen, and some professional associations), the Social Security Insurance Organization (SSO) covering all people employed in the formal private sector and their dependents, and the Armed Forces Medical Services Insurance Organization (AFMSIO) [8],
2. **Institutional organizations:** It includes about 17 funds such as the municipality, the Petroleum Industry Health Organization, the National Broadcasting Organization, banks, and other organizations. Each organization provides required insurance services for its employees individually as a fringe benefit [9], and
3. **Commercial organizations:** Among them are Iran, Asia, Alborz, Mellat, Pasargadae, and Atieh Sazane Hafez Health Insurance Organizations, of which the latter often operates in the form of voluntary supplementary private insurance [10–15].

In recent years, there has been growing concern about problems attributed directly or indirectly to fragmentation in the Iranian health financing system. Among these problems are inequity in health service utilization and financial protection among different groups of people [13, 16]; high out-of-pocket expenditures [12, 13, 17]; high occurrence and intensity of catastrophic health expenditures [18, 19]; low financial protection against health services for insured people [10, 20]; population coverage duplication [10, 12]; failing to reach universal health coverage; lack of transparency and no reliable data in population coverage; per capita health expenditures, and contribution rates [10]. Such problems have urged policy makers to pass the 2010 Act to merge all the existing health insurance funds (basic health insurance schemes and institutional funds) into MSIO, aiming to create a single health insurance organization [9, 21].

### Objectives of the study

This study aims to investigate what potential advantages and disadvantages merging health insurance funds in Iran may bring to the health insurance system in particular and the health system in general, in Iran. The merging health insurance is a context-sensitive issue, and exploration of its advantages and disadvantages has received scant attention in qualitative analyses in the research literature. Thus, this study may provide valuable insights into moving toward a decision on the merging health insurance in developing countries such as Iran. Lessons from this study can be applicable for policy makers from other countries, especially those with low and middle incomes, trying to merge existing health insurance schemes in order to strengthen risk pooling.

## Methods

This qualitative study was conducted in 2014–2015. This paper is part of a larger study about “analysis for policy” of merging social health insurance (SHI) funds in Iran.

### Participants

A purposeful sampling with maximum variation was used to obtain representativeness and rich data by including a wide range of extremes. As the use of maximum variation sampling is a well-established approach to limit the possibility of selecting narrow or few cases from one with wide variation [22]. To develop an initial list of stakeholders (including organizations, institutions, political groups, etc.), a number of qualitative Iranian papers on health financing, health insurance, and consolidation articles of the SHI Funds Law adopted in the Fifth Five-Year National Economic, Social and Cultural Development Plan, were reviewed. Furthermore, the advice of members of the research team familiar with the context of the health insurance system in Iran was addressed. Table 1 shows the list of different stakeholders from which the interviewees were selected.

**Table 1:** The main stakeholders of merging health insurance funds in Iran

Setting	Organizations, Institutions, political groups, and individuals	No.
Health Insurance System	The Ministry of Cooperatives, Labor and Social Welfare including the High Council of the Health Insurance	**3**
	Iran Health Insurance Organization	**16**
	Social Security Organization	**8**
	Armed Forces Health Insurance	**2**
	Imam Khomeini Relief Committee	**2**
	Insurance companies	**3**
	Municipality of Tehran	**2**
	Petroleum Industry Health Organization	**3**
Ministry of Health and Medical Education (MoHME)	Health Technology Assessment (HTA), Standard and Tariff Office	**3**
	Supreme Council for Health Policy Making	**2**
	Deputy for Medical and Curative Affairs	**1**
	Former Minister	**1**
	Budget and Performance Monitoring Office	**2**
Health Care Providers	The Medical Council of Islamic Republic of Iran	**3**
	Iranian Medical Council	**2**
	Hospitals (staffs responsible for reviewing medical records according to different principles of different health insurance funds for reimbursement)	**2**
	Society of Radiology	**1**
	Pharmacists Association	**1**
	Association of General Practitioners	**1**
Supervisory/regulatory Organizations	Planning and Budget Organization	**2**
	Parliament Research Center	**1**
	Parliament Representatives	**4**
Medical Sciences Universities	Academician familiar with Iranian health system and health financing concepts	**2**
***Sum***		***67***

As members of the research team were quite familiar with the Iranian health financing system and the main policy actors, an initial list of key informants for each stakeholder was selected by the research team purposefully for doing interviews. Key informants with outstanding experience, work, research, and knowledge background in the area of Iranian health financing were selected. The interviewees were mainly the top managers and key policy actors. Of course, in 9 cases, interviews were conducted with front-line employees in different departments of health insurance organizations and hospitals to get more detailed information and more profound insight into the challenges, benefits, and drawbacks of merging health insurance funds in Iran. Overall, sixty-seven, face-to-face interviews were conducted. Out of sixty-seven interviewees, sixty-two were male, and five were female. Participants’ age ranged from 33 to 80 years. Other additional stakeholders and key informants were identified through snowball sampling and information derived from the analysis of interviews and relevant documents.

Interviews continued until there was no more key informant and stakeholder and also no more new ideas were identified, and saturation was reached. The documentary review in line with interviews was used as a supplementary source of data collection to investigate the aims, intentions and reasons for moving toward to merging health insurance funds and identifying the potential drawbacks and merits of it. The principles of the Five-Year National Economic, Social and Cultural Development Plans; the 20-Year National Vision; the main laws and reforms in the history of developments in Iranian health insurance; TV programs, TV interviews, reports, declarations, newspapers, and proceedings of the Iranian Parliament on the Law of Consolidation were among the documents examined and analyzed.

### Conceptual framework

A semi-structured interview guide (see Additional file 1) was developed for this study to conduct the interviews. For the study, to develop the interview guide questions and finding an appropriate conceptual framework to cover all aspects of merging and to organize the findings, a conceptual framework was derived from the World Bank [23]. The World Bank framework includes eight elements to design and establish a health insurance system: feasibility of insurance design, financing mechanisms, population coverage, basic benefit package, provider engagement, organizational structure, operational processes, and monitoring and evaluation. As the authors believed that this framework was comprehensive enough to cover the main aspects of the health insurance system, it was selected to classify the advantages and disadvantages of the merging of health insurance schemes in Iran. The interview guide was pilot tested by doing three introductory interviews.

### Data collection

At the beginning of each interview, by explaining the purpose of the study and ensuring the confidentiality of interview content and anonymity, the interviews were taped with one voice recorder. Interviewees were free to choose where they liked to be interviewed, which in all cases, interviews were done in the workplace of the interviewees. Besides the researcher and participants, no one else was attended during the interviews. In three cases, the participants did not allow voice-recording (they were afraid as the topic was political); therefore, notes of the main points were taken down. Also, field notes were made during and/or after the interviews to get the most out of the interviews. The minimum, maximum, and average times of interviews were 10, 120, and about 50 min, respectively. In seven cases, the interviews lasted for two or three sessions because interviewees were busy trying to finish the interview in one session. Some of them were eager to give more information, and in some cases, the interviewer had to refer again later for more details or to complete omitted information. In the case of document review, the list of related documents, and their source for the collection were identified. Websites of organizations including Majlis, MoHME, Iranian Medical Council, and Health Insurance organizations were reviewed and related in print documents were also collected in person.

### Data analysis

Content analysis using the ‘framework method’ was used to analyze the qualitative data. It is worth mentioning that this method can also be applied in deductive, inductive, or combined types of qualitative analysis [24]. The five-stage process of qualitative data analysis was done: understanding (familiarization), identifying a thematic framework (thematic), coding (indexing), charting and mapping, and interpretation [25]. All the interviews were done, transcribed, and initially indexed by one author (MB). Interviews were analyzed with the World Bank framework (both inductive and deductive approach) using MAXQDA12 software.

### Trustworthiness

For assuring the quality of results, we employed four trustworthiness criteria suggested by Lincoln and Guba [26]. Credibility was met with a prolonged engagement whereby the principal investigator (MB) continuously worked nearly 12 months with the qualitative data. Furthermore, member-checking validation was used by delivering some transcribed interviews to the respective participants. They then asked them to ensure that there is a good correspondence between their findings and the participants’ perspectives. To improve credibility, particularly for this study, we focused on the contrary and opposite cases to provide a comprehensive picture derived from the pros and cons of merging. The research team search for and discuss elements of the data that do not support or appear to contradict patterns or explanations emerging from data analysis and deviant cases in findings were incorporated in the analysis process until it can explain or account for a majority of cases. The transferability and reflexivity of our qualitative findings have been enhanced by the maximum variation sampling technique and the detailed description of the health insurance context in Iran. Dependability of the research was assured by an auditing approach in which the VYF accompanying by an external auditor engaged in critical comments in the coding process and analyzing of transcribed interviews as well as cross-checked the data we collected. To increase confirmability, we employed a methods triangulation approach, including document review, interviews with key informants, and other informative sources to check out the consistency and complementary of findings generated by different data collection methods.

## Results

The advantages and disadvantages of merging health insurance funds derived from the interviews were classified in the following categories: stewardship, financing, population, basic benefits package, structure, operational processes, and interaction with providers. The key advantages and disadvantages of merging health insurance funds in Iran obtained from the interviews are presented in Table 2.

**Table 2:** The advantages and disadvantages of merging health insurance funds in Iran derived from the interviews

Theme	Sub-theme	Pros of Merging Health Insurance Funds in Iran	Cons of Merging Health Insurance Funds in Iran
**Governance/Stewardship**	The accountability of health insurance regarding insureds’ needs and demands	• Increasing the accountability of the health insurance system regarding how to generate and use financial resources to meet insureds’ needs by creating a single health insurance fund	• The risk of objection by workers as they may consider the social security organization responsible for their treatment • The bad memory of the past related to the Ministry of Health and Medical Education (MoHME) for not being responsive to how and where it spent the health premiums of the social security organization’s beneficiaries • Reducing accountability by the creation of a monopoly in the health insurance system like what happened in the car industry in Iran
	The control of health care expenditures	• Multiple health insurance schemes, leading to an increase in inflation and purchase of health services at higher prices • Reducing the cost of the health insurance system by eliminating duplication in insurance coverage and solving the problem of using health insurance cards by those without insurance coverage • Cost control by implementing strategic purchasing • Better supervision of health care providers by centralizing their profiles in a single database, reducing fraud, and controlling the volume of provided health care services	
	The power of health insurance supervision	• Providing a better chance to enhance supervision by reducing delay in payment and timely reimbursement of health care providers • Improving the quality of health services by providing better supervision and higher purchasing power • Improving the supervision by adding personnel merged into the regulatory area	• The lack of chance for enhancing the supervisory role of health insurance schemes as they have no real power to supervise the quality of health services at the current situation • Not envisaging the task of supervision for health insurance in the Merger Law in the Fifth National Economic, Social, and Cultural Development Plan Act • Reducing the control of other health insurance schemes on how the Iran Health Insurance Organization (IHIO) spends health insurance premiums • The risk of increasing supervision costs for other health insurance schemes to examine how their premiums are spent by IHIO (the same experience happened in 1983, when a commission was established in each province to supervise the quality of treatment for the social security organization’s beneficiaries provided by the regional health centers of the Ministry of Welfare and Wellbeing) • The risk of not being able to supervise and control the national insurance scheme as it may become so powerful
	The health system management	• Easier alignment of a single insurance scheme with the policies of MoHME • Easier implementation of clinical guidelines • The interaction of MoHME with a single insurance scheme with a single set of instructions • Putting an end to different policies issued by different insurance schemes in dealing with health problems (e.g., updating the table for prices of medicines • Eliminating the challenge of unifying health insurance schemes with different regulations as an obstacle to implement health reforms such as a family physician program • Improving the health financing equity by setting the same coinsurance rates for different population groups	
	The transparency of health information and policy making	• Providing a more reliable planning and policy making strategy by centralizing health insurance information in a single data bank • Making a more precise prediction of financial resources and annual budget required for the health insurance system by providing transparency in per capita health insurance expenditures and eliminating duplication in population coverage	
	Policy making and stewardship in the basic health insurance system	• Increasing the power of the health insurance system to formulate and implement health policies • An easier and faster process of making health policies by creating a single scheme • Organizing different health policies and decisions issued by different health insurance schemes by merging	
**Financing**	Health insurance premiums	• Reducing premiums as a result of reducing costs	
	The ability to create new resources		• Other health insurers may lose motivation to collect premiums and transfer them to IHIO (the unwillingness of the social security organization to collect insurance premiums from workers)
	Per capita premiums and actuary calculations	• More reliable calculations of per capita premiums by removing duplication in insurance coverage	
	Patient risk pooling	• Equity in risk pooling for all population groups as rich schemes have no contribution in the whole risk pooling	• Not considering merging as a solution to improve risk pooling because more than 90% of the whole population is under coverage of IHIO and SSO
	Financial inflows and outflows	• Easier monitoring of accounting and financial processes • More transparent and stable insurance financial inflows and outflows as a redult of merging health insurance funds	• More transparent financial flows in the current fragmented situation as each scheme has its own separate financial flows • Keeping separate financial accounts for sub-funds under IHIO for higher transparency in revenues and expenses
	The estimation and management of financial resources in the health insurance system	• Easier estimation of the amount of required financial resources for the following year by creating a single insurance system • Better management of health insurance premiums by pooling them in one place • Saving the public budget by removing coverage duplication • Being able to define a new role for health premiums by centralizing them in a single fund • Obtaining more financial support from the government for basic health insurance	
	Patient financial protection	• Paying lower premiums for the treatment of low-income people as a result of merging • Expanding benefits packages by saving resources • Better controlling the high cost of health services • Reducing out-of-pocket payments by purchasing health services at lower prices with single insurance	• The need to increase premiums and resources to reduce people’s payments
	The financial stability of health insurance	• Increasing the financial viability of the whole health insurance system by using single insurance	• The appropriate size of IHIO and SSO at the current fragmented situation (IHIO and SSO are big enough and there is no need for consolidation)
	Saving financial resources	• Increasing financial resources and improving health benefits packages by reducing administrative costs • Organizing financial resources spent by the government as the employer for different population groups by merging	
	Equity in the distribution of subsidies in the health insurance system	•Difficulty in providing all population groups with the same share of governmental subsidies in a fragmented health insurance system	
	A more efficient use of health insurance premiums	• Imposing expenditures by non-contracted health care providers at the current fragmented system • Paying for luxury and expensive private hospitals’ health services at the current fragmented system • Relying on fee-for-services payment method without having effective supervision and control on the amount and quality of provided health services at the current fragmented system • Using health premiums more efficiently by eliminating induced demands and moral hazards Paying higher prices for health care services by well-off and small insurance schemes due to not having any chance to follow strategic purchasing in the current fragmented situation	Having no chance to implement real strategic purchasing unless setting real medical tariffs according to real prices
**Population**	Reaching universal coverage	• Fragmentation in the health insurance system as a barrier to reach universal coverage in the three areas of population, health services, and financial protection	
	Population coverage duplication	• Eliminating duplication in population coverage • Eliminating inequity in access to health services due to health insurance coverage duplication; currently, due to this issue, different groups of people use different benefit packages with different services and financial protection belonging to various health insurance schemes) • Centralizing health profiles and health expenditure profiles of beneficiaries in a single database • Reducing the misuse of multiple health insurance cards by health service providers and patients	
	The number of insured people	• Providing precise and reliable statistics about the number of insured people by creating a single database and removing duplication	
**Basic Benefit Package**	The focus of health provision (primary health services or hospital-based services)	• Neglecting prevention and public health services as a result of fragmentation • Providing a better chance to focus on and pay more attention to public health and preventive services by creating a single health insurance	
	The distinction between basic and supplementary health insurance	• Eliminating the current interference between basic and supplementary health insurance (in Iran, according to the national health laws supplementary are supposed to cover only those health services not covered by basic health insurance funds)	
	Equity in the basic benefits package	• Strengthening benefits packages for underprivileged groups by setting the same benefits package for all population groups • Putting an end to discrimination and provision of generous health services for privileged groups • Currently, various funds react differently to changes in prices of medicines and medical equipment, and also, to changes in the basic benefits package. In the case of high fluctuation or high inflation in prices, the health insurance funds may update the prices they cover in different time lags which causes different population groups pay different amount of out-of-pocket expenditures for the same medicines or medical supplies.	• The lack of justice due to poor infrastructure and health care facilities in villages and deprived areas • No justification for providing the same benefits to all insureds (different population groups with different health needs, expectations, and affordability)
	The satisfaction of insured people	• Increasing the satisfaction of insured people by strengthening and upgrading the basic benefits package • Resolving dissatisfaction among the public as a result of disparity and injustice in benefits packages in the current fragmented situation • Increasing satisfaction by improving competition among health service providers • Providing equal access to health care for all insured people	• Increasing dissatisfaction among population groups enjoying generous benefits package in the current fragmented situation • Concerns about sharing privileges with other groups of insured people (e.g., sharing free-of-charge health services in health centers and hospitals belonging to SSO)
**Structure**	Creativity, innovation, and dynamism in insurance	• Increasing the dynamics and creativity in devising new mechanisms to run activities with creation of single insurance (no chance to use creativity made by other health insurance funds)	• Killing creativity, as, currently, each health insurance fund attempts to improve the quality of its own services
	Administrative and overhead costs	• Reducing administrative and overhead costs by removing parallel structures of insurance in all the provinces (each social health insurance scheme has its own headquarter in each province) • Reducing administrative costs by reducing the number of employees and top managers • Reducing personnel costs in the long run as all departments and employees in each health insurance fund with the same job description would be merged and there would be no need to recruit the same number of personnel as before • Reducing supervisory costs by unifying the content of contractions between health insurance schemes and hospitals with health care providers	• No tangible reduction in administrative costs in the short run due to political resistance against downsizing • Emphasis on reducing administrative costs as an inadequate target for insurance merging
**Operational procedures**	The monitoring and supervision of health care providers	• Better recognizing drug interactions • Resolving the issue of abuse and fraud by health care providers in the current fragmented situation • Easier monitoring and control of providers by creating a single central profile for each provider	
	Administrative and financial instructions	• Unifying financial and administrative regulations and instructions and reducing the complexity of different regulations for health care providers • Unifying operational instructions to enhance the the whole health insurance system performance • Formulating new regulatory instructions for the private health sector • Formulating new regulations to organize a supplementary health insurance market by creating a strong single basic health insurance fund	
**Interaction with providers**	Competition in the health system	• Creating competition among providers by creating a single-payer system • Improving the performance of health insurance system by merging as there is no real competition among health insurance funds in the current fragmented situation	• Eliminating competition between basic health insurance schemes
	The control of providers	• Changing providers’ behavior by making financial leverage • Reducing costs imposed by non-contracted providers • Resolving the issue of the non-compliance of private health care providers due to the fragmented health insurance system • Resolving the issue of the need for small insurance to contract with the provider at a higher price due to fragmentation	• The risk of putting health care providers into difficulty by misusing the power of monopsony and not fulfilling commitments
	Bargaining power	• Increasing the bargaining power of insurers by moving from a *multiple-payer* system to a single-payer system	• The lack of authority of insurance funds to bargain and determine medical tariffs
	Strategic purchasing	• Boosting strategic purchasing, as each insurance fund follows a certain approach in the current fragmented situation • Increasing fund flexibility by pooling financial resources • Enhancing strategic purchasing by improving financial resources and faster reimbursement • Improving strategic purchasing by avoiding higher payments by small insurance funds • Providing better financial resource management by creating a single-payer system and mass purchasing • Better controlling the private sector by strategic purchasing	• The lack of need to subject strategic purchasing to single insurance • The inability to buy health services at lower prices due to low and unrealistic tariffs • The unavailability of insurance at affordable prices and single-rate sales by MoHME
	Reimbursement to the provider	• Making timely reimbursement of providers	• The risk of putting more financial pressure on providers in case of delay in reimbursement by the single-payer (currently, providers are reimbursed by different insurance funds with different time tables) • The risk of putting more financial pressure on providers by following strict financial regulations
	Modifying the payment system	• Providing a better opportunity to design and implement new payment methods by a single insurance fund	
	The interaction of health insurance system with health care providers and hospitals	• Formulating new single instructions for providers and finishing different intricate details of regulations of multiple insurance funds • The lack of need to appoint different staff to audit different medical health records belonging to different health insurance funds • Unifying the regulations of purchasing health care services from providers • Unifying the details of basic benefits packages (using the same services, using the same prices for pharmaceuticals and medical supplies, using the same reference pricing, using the same exceptions, using the same coinsurance rates for different services and patients, etc.) for all hospitals • Reducing transaction costs and preparing different insurance bills	
	Organizing private health sectors	•Resolving the issue of low and delayed reimbursement provided by basic health insurance funds	

### Stewardship

In 2004, by creating the Supreme Council of Health Insurance (SCHI) under the Ministry of Cooperatives, Labor and Social Welfare, a purchaser-provider split occurred in the Iranian health system to move toward strategic purchasing and boost competition among health care providers. According to studies, after the split, lack of coordination between two the ministries caused new challenges for Ministry of Health and Medical Education to devise and implement health reforms without control of financial resources [27]. Apart from this, fragmentation in health insurance caused each insurance scheme to behave differently and follow different health policies, and also, implement policies issued by SCHI differently. According to the interviews, merging can solve these challenges to a great extent. According to the findings, merging and creating a single national health insurance system can provide a situation, in which it is easier to control total health care expenditures as well as to formulate and implement more reliable health policies for the health system.

*"…I'm really tired of attending meetings of the family physician and referral system. I've probably attended more than 50 large meetings regarding launching a family physician and referral system myself. We saw that the IHIO representative wanted something different, the SSO' agent spoke differently, and the AFMSIO's representative said something else. If I, as MoHME, wanted to implement a family physician referral system, whose opinion should I accept? ..." (P2, a policy maker in the health insurance system)*

*"…The Social Security Organization doesn’t implement whatever approved by HCHI or implement them with delay…" (A parliamentarian, Nabz, a TV program about the Iran health system problems)*

*"…wherever monopoly is formed, accountability is reduced. Since all people have to get their services only from one organization with the same quality and quantity, this causes reduced responsiveness as this organization has no competitors. … the same thing happened to our car industry as a result of monopoly. …" (P20, a manager in one of the health insurance organizations)*

### Financing

Fragmentation in health financing in Iran has caused each health insurance scheme to follow its policies. In the long run, it led to differences in contribution rates, out-of-pocket payment rates, and coinsurance rates; different levels of financial protection; and an uneven distribution of public subsidies among different insured groups. Apart from reducing inequities, the interviewees believed that merging could improve the collection, management, pooling, and allocation of financial resources to purchase health services for beneficiaries.

*"… About 23 million rural citizens are covered freely by IHIO; the government pays for them. Is there this advantage for workers? Aren’t they Iranian? isn’t it discrimination?" (Nabz, a TV program about the Iran health system problems, a key policy actor)*

*"When you have duplication in coverage, more public budget is spent. It means that the government pays twice as the employer for the same group of population…" (P38, a policy maker in one of the supervisory organizations)*

### Population

In the population area, the following subjects were the main topics, on which merging of health insurance funds may have positive or negative impacts: extending coverage for those without health insurance and removing the problem of duplication in population coverage.

*"… When the supervisory and legislative agencies requested (health insurance schemes for) the number of insured people, adding the numbers together, we saw that the total number was larger than the whole population of the country, and at the same time, we had ten million people uninsured. …" (P38A, a policy maker in one of the supervisory organizations)*

*"… One of the merits of merging is unifying the population's information. According to the Iranian census, 77 million people are known; when you combine and unify all health insurance databases, it will make those people without insurance coverage clear. Why is making it clear impossible now!? Since they are scattered in 17 databases; merging makes it clear who has several insurance cards and who has no coverage …" (P26, a parliamentarian)*

*"… The fragmentation and duplication of health insurance coverage make it difficult to calculate the per capita expenditures accurately. As a result, the computation of insurance premiums will be blurred. …" (P17, a former manager in one of the health insurance organizations)*

### Basic benefits package

The first advantage mentioned by most of the interviewees in this aspect was providing an equitable basic benefits package for all Iranians. According to the interviews, high inequity in benefits packages under the coverage of insurance schemes has led to high dissatisfaction among people, which is unacceptable and against national values and constitution. The existing discrepancies between various demographic groups in terms of types of health services that they can receive, amount of financial compensation offered for each health service, and number and types of health care facilities (public or private health sector) where they can receive their services will be abolished by providing a single health insurance system for all demographic groups.

*"… We (health insurance experts in Iran) believe that we are moving toward public health-based services (conserving health status); but, what we are doing now is providing hospital-based services…" (A manager in one of the Health Insurance Organizations)*

*"…the main focus of some health insurance funds is on business, and not on health…" (A parliamentarian, Nabz, a TV program about the Iran health system problems)*

### Structure

The interviewees believed that main advantages in this section were related to the reduced administrative and overhead costs as a result of eliminating parallel structures of insurance in the provinces of Iran and reducing the number of top managers and employees.

*"… all insurance funds have their own offices in different provinces. Different insurance companies have their own offices, general directors, secretaries, cars, traveling costs, seminars, and so on. …" (P7, a parliamentarian)*

*"… Meanwhile, these 18 companies have created their specific funds; they pay high salaries to their CEOs and boards of directors. If they are merged, instead of having 18 boards of trustees including 60 to 70 top managers, we face one board of trustees. …" (A parliamentarian, Nabz, a TV program about the Iran health system problems)*

### Operational processes

Eliminating different instructions applied by health insurance funds to review claims and provide better supervision and management of health care providers by merging their health profiles in a single database was the main advantage stated by the interviewees.

*"… Overall, the fragmentation of insurances has many challenges. One of the challenges is that there are different guidelines and rules. It confuses providers; it even confuses the medical association; regulations existing in IHIO are different from those in the social security organization (SSO), with one covering different and more health services compared to the other one. The depth of coverage of the armed forces is the most. A physician must make several lists for different insurance schemes, which means that both the physician and his secretary should put more time to prepare them; this increases administrative costs. …" (P18, a policy maker in the health insurance system)*

*"… when I [pharmacist] buy a drug, I get a fee list from the IHIO website, and also, I have to check out the fee list of SSO. For example, for Albumin, IHIO charges 31,000 Tomans, Imam Khomeini Foundation charges 38,000 Tomans, and Social Security charges 27,000 Tomans. …" (P5, a manager in one of the health insurance organizations)*

### Interaction with providers

Merging and creating health insurance schemes can influence the interaction with health care providers in various ways. Merging can positively influence the following areas: competition among health care providers, strategic purchasing and supervising health care providers, reimbursement and moving toward new payment methods, and the principles of contraction with providers.

*"… The next problem is that medical fraud is easier to occur in a fragmented health insurance context because providers' medical profiles are not centralized in a single database. Someone may misuse an insurance scheme, and it takes time to recognize this fraud based on other health insurance schemes. …" (P17, a former manager in one of the health insurance organizations)*

*"… When the profile of a physician is centralized in one database, you can see how much drugs or paraclinical diagnostic tests they have prescribed, and you can supervise them better. Doctors are intelligent; they obey health insurance schemes with strict rules, but they may play games with other schemes. With single insurance, doctors are reimbursed by a single-payer system. So, you can conduct taxation affairs easier. The current fragmented situation is better for those doctors who want to avoid paying taxes" (P18, a policy maker in one of the health care provider associations).*

## Discussion

The successful merge of health insurance funds like other health policies is dependent on identifying and dealing with a wide range of different factors rooted in the contextual factors in each country. This issue is of paramount importance, particularly in developing countries that are faced with limited sources and different structural, institutional, and political conditions. This study aimed to realize the advantages and disadvantages of moving toward a decision for the merger of health insurance funds. The results of this study indicated that there are different positive and negative consequences for merging health insurance funds in Iran, which are categorized into seven categories, including governance/stewardship, financing, population, basic benefit package, structure, operational procedures, and interaction with providers. These themes are subdivided into thirty-seven sub-categories representing a wide range of different policy aspects to pay close attention to dealing with the merging of health insurance funds.

It is worth to specify that what kind of problems the consolidation of health insurance schemes can solve in the health system and health financing area. Also, the clear explanation of potential achievements can be effective in supporting the implementation of the program and reducing the resistance by opposing actors.

According to the results, combination and changing financial flows, and the increase in the power of the health insurance system, can improve the equity in health financing. Although health equity improvements cannot be attributed only to the consolidation or reduction in the numbers of health insurance funds, consolidation can be effective to a great extent in reducing the current inequity. For instance, out-of-pocket share in Turkey accounted for 19% of the total health costs 3 years after the implementation of the program, which was considered fairly low [28]. In South Korea, the same contribution rates were developed for all the self-employed across the country as a result of the merger [29].

Bigger funds will improve the economies of scale, which will, in turn, maximize the benefits provided for the members. According to the findings, reducing administrative costs by reducing the number of health insurance organizations in all provinces and reducing the number of top managers and employees can be attributed to the merger as one of the first advantages which comes to mind. Similarly in Korea, after the merger of the regional funds of the self-employed with the fund of government employees and school teachers in 1998, 227 insurance funds of the self-employed and 19 funds of the government employees were reduced to 162 regional funds, and the number of personnel was reduced from 10,849 to 9073 in December 1999 [27]. Reducing administrative costs has been emphasized in international literature as one of the advantages of having fewer risk pools [29–32].

The international experiences show that the single-payer system is more powerful and efficient in controlling the total health care expenditures [29, 30]. Regarding risk pooling efficiency and financial stability, the single-payer is more preferable [33]. The collection of contributions will be integrated with other social insurance funds as a result of merging. In addition to the improvement of equity in contributions and reduction of administrative costs [29], the single-payer system has more bargaining with providers through creating a monopolistic purchaser [29]. The single insurance has the capacity and inclination to purchase medical care cautiously, which will improve the efficiency of the new system [29]. Also, the single insurance system will increase the competition among providers, since the single insurance is the only payer and provides providers a free choice [30].

The single insurance system can enhance insurance packages and extend the coverage in favor of the poor and the members of weaker insurances. For instance, it is expected that in Indonesia, the uniform service package for civil servants and the private sector employees will create better clarity, equity, and understanding of the package for providers and members [30].

It is also worth mentioning that the experience of other countries moved toward merging shows that the single insurance system can provide an opportunity to highlight some neglected health insurance policy decisions previously in the fragmented health insurance system at a national level [29]. According to the interviews, currently, the main focus of health insurance schemes in Iran is on hospital-based services. Despite the fact that PHC in Iran is financed by the government and provided by the district health network, the interviewees criticized the current situation, believing that the health insurance system failed to address public health and preventive services and also to preserve health at the outset, which, in turn, led to high expenditure on health care. In the current fragmented situation, health insurance schemes struggle to cover more secondary and tertiary health services, while merging can help to focus on public health services and prevention as a policy to move towards financial resources management by controlling health care expenditures in the long term.

Typically, one of the consequences of the multiple insurances is that despite existing different health insurance organizations alongside each other, a part of the population is not covered by any insurance for different reasons [34, 35]. Creating a single health insurance database would make it easier to eliminate duplication and identify those who have no coverage, which will pave the way toward reaching universal coverage.

Besides the positive effects, merging may cause unpredicted side-effects in the health system in the short and long run, which need to be mitigated. A single-payer may reduce efficiency due to increasing the bureaucracy and decreasing responsiveness; however, in Iran where people do not have real right to choose between different funds (people are assigned to different insurance funds according to their job status or where they live), the efficiency lost as a result of merging may not be significant [29]. In a single-payer system, the insured does not have the chance to choose and changing the insurer when the health services are not satisfactory, which can lead to dissatisfaction, especially in affluent families. However, we need to know that the right to choose the provider is much more valuable and significant than the right to choose the insurer. Insurers are only payers and have little effect on the process and outcomes of the treatment. In the single-payer system, the free choice of the provider and increased competition among providers can increase the satisfaction of the beneficiaries [30, 32].

According to the consolidation law in Iran, it is supposed that by merging, all health insurance schemes are responsible for collecting their contributions and allocating their share to the IHIO. Some of the interviewees were concerned about SSO’s unwillingness to collect premiums as actively as before, as the financial resources will not be managed and spent by the SSO. In South Korea, health experts are concerned that as the financial resources are going to be shared with the whole population, collection of the contributions from the self-employed after merging may not be done as actively as before [29].

In contraction with health care providers, interviewees mentioned that currently, each health insurance scheme follows its regulations for contracting and also for reviewing claims and reimbursement. Apart from increasing complexity, hospitals have to appoint specific employees to deal with different regulations issued by different health insurance schemes, which increase administrative costs. Creating a single insurance scheme would make it much easier and simpler to work with one insurance scheme and one set of rules. Since the second half of 2019, IHIO has recently started to launch new projects to handle claims and also manage the referral system based on an electronic system. Although such a good initiative increases the speed, accuracy, and effectiveness of claims handling and referral systems, other major health insurance organizations such as SSO and AFMSIO use non-electronic systems that make it more difficult for health care providers to work with different systems of health insurance.

Interviewees mentioned contradictory ideas about the impact of merging on the reimbursement process. Merging may improve or even worsen the time of reimbursement. Some indicated that health insurance schemes currently behave differently in terms of the time of payment and the amount of payment for the same health services. Health care providers express their concern about how merging is going to change the process of payment. Health care providers said they are currently paid by several schemes on different periods of time, in the case of delay in payment by one or two schemes, health care providers resort to other schemes with on-time reimbursement. Nevertheless, they worry that by creating a single-payer, in the case of delay in reimbursement, the financial security of health care providers would be jeopardized.

### Study strength and limitations

A vital strength of the present study is that we interviewed with a maximum variation of stakeholders with confirming and disconfirming perspectives about the advantages and disadvantages which may enhance the trustworthiness of results. This helps clarify the path of moving toward the implementation of consolidation law in Iran. This study was conducted after passing the Consolidation Law in Iran when the government was mandated to implement the law. Whereas no action has yet been taken, it provides a natural policy environment in which the expectations of the stakeholders in the implementation of the law, as well as their conflicting interests, could be better captured in the study. Of course, these data must be interpreted with caution because it is not conclusive which advantage or disadvantage mentioned by interviewees may occur in the real-world after the implementation of the law. Furthermore, in some aspects such as stewardship or financing, there were contrasted and opposite opinions mentioned by informants about the advantages or disadvantages of the consolidation. It is not clear which stakeholder correctly points out the advantages or disadvantages or gives a more accurate interpretation of what will happen in reality. At the same time, some of the interviewees may have had limitations in expressing their opinions due to political consideration and their day-to-day responsibilities and institutional positions. This means that we also contend that key stakeholders interviewed might have been affected by social desirability and position bias, which means that they may have described what they thought we want to hear, rather than the reality. Some of them may have provided politically acceptable and satisfactory responses concerning their roles and responsibilities. In the deal with these problems, we used triangulation of data collection methods, including interviews, document analysis, and some mass media sources, and compared people with different viewpoints, opposing and supporting the policy. Also, triangulation of sources and as well as providing ample opportunities to the interviewees to express their deep understandings of the context, raised the credibility, confirmability, and reflexivity of the results. Last but not least, these findings may be somewhat limited by subjectivity. Although we used different data sources and analyzed the data by peer debriefing to enhance the trustworthiness of the results, the interpretations may persist subjectively, and our results cannot claim a whole truth. As the philosophical research paradigm is a constructive approach rather than a positivism one, this situation is unavoidable and is defensible.

## Conclusions

The present study was designed to investigate different stakeholders’ viewpoints about the potential advantages and disadvantages of health insurance funds consolidation in Iran, a law passed but not implemented. Both proponents and opponents tended to highlight potential benefits and drawbacks of merging in a way to encourage or hinder taking steps toward implementing the law. The act of proponents could have been effective in preventing the implementation of the law in Iran as the opponents tried to signalize the drawbacks and disparage the merits of merging or attribute the current problems in the health insurance system to other things rather than fragmentation. The most prominent finding to emerge from this study is that consolidation implementation in Iran may be influenced by a wide range of merits and drawbacks in governance/stewardship, financing, population, benefit package, the structure of health insurance, operational procedures, and interaction with providers. This study helps to inform policy makers in low resource settings about the potentially expected benefits and detriments when moving toward the implementation of this law. In summary, the following benefits were raised as the main benefits of merging health insurance funds in Iran: controlling total health care expenditures; centralizing the profile of providers in a single database; reducing fraud and controlling the volume of provided health care services; making hospitals interact with single insurance with a single set of instructions for contracting, claiming review and reimbursement; reducing administrative and overhead costs and reducing the number of employees. Following consequences were mentioned as main potential drawbacks of merging health insurance funds which should be addressed by the policy makers carefully: the social security organization’s unwillingness to collect insurance premiums from private workers actively as before; increased dissatisfaction among population groups with generous benefits package; risk of financial fraud and corruption due to gathering all premiums in a single bank; and risk of putting more financial pressure on the providers in case of delay in reimbursement by the single-payer.

All these results are highly context-sensitive, and there is a trade-off between benefits and drawbacks before and after consolidation. Thus, policy makers should act as brokers taking into account the contextual factors and adopting tailored policies to maximize the potentiality of realizing the benefits and devise precautions to minimize the likely drawbacks of consolidation.

## Supplementary information

**Additional file 1.** Interview guide. The Interview guide includes the open-ended questions which were asked from interviewees.

## Data Availability

All raw data have been prepared in Persian (not English). However, the corresponding author will gladly provide any supporting materials upon request.

## Abbreviations

AFMSIO: Armed forces medical services insurance organization
IHIO: Iran health insurance organization
MHIF: Merging health insurance funds
MoHME: Ministry of health and medical education
SCHI: Supreme council of health insurance
SHI: Social health insurance
SSO: Social security organization
