# Supplementary material for: What are the potential advantages and disadvantages of merging health insurance funds? A qualitative policy analysis from Iran
Source: BMC Public Health. 2020 Aug 31;20:1315. doi: 10.1186/s12889-020-09417-7 (PMC7457517; doi:10.1186/s12889-020-09417-7)
Supplement: Supplementary file 1 — Additional file 1. Interview guide. The Interview guide includes the open-ended questions which were asked from interviewees. [file 12889_2020_9417_MOESM1_ESM.docx]

Additional file 1

**Interview Guide**

1. In your opinion, what kinds of positive and negative consequences can merger of health insurance funds in Iran generate in the ***“health system in general”***?
2. What kinds of positive and negative consequences do you think the merger of health insurance funds in Iran can bring about in the ***“health insurance system in particular”***?
3. How can merging health insurance funds in Iran influence the ***process of health policy making*** positively or negatively? *Please explain*
4. In the Iranian health insurance system, what kinds of problems can be solved in the area of defining ***basic health insurance benefit package*** as a result of merging health insurance funds? What challenges may rise vice versa?
5. In the Iranian health insurance system, what kinds of problems can be solved in the area of ***health financing*** as a result of merging health insurance funds? What challenges may rise vice versa?
6. In the Iranian health insurance system, what kinds of problems can be solved in the area of ***population coverage*** as a result of merging health insurance funds? What challenges may rise vice versa?
7. What are the potential advantages and disadvantages of merging health insurance funds in Iran in the area of Iranian health insurance system’s ***structure***?
8. What are the potential advantages and disadvantages of merging health insurance funds in Iran in the area of Iranian health insurance system’s ***operational processes***?
9. How can merging health insurance funds in Iran affect the quality of ***interactions between purchaser (health insurance funds) and provider*** (hospitals and health care providers) and positively or negatively?
10. If there are other overlooked merits and drawbacks in other areas please explain.
